# Primary care professionals’ experiences during the first wave of the COVID-19 pandemic in Greece: a qualitative study

**DOI:** 10.1186/s12875-021-01522-9

**Published:** 2021-09-03

**Authors:** Emmanouil Smyrnakis, Despoina Symintiridou, Martha Andreou, Michael Dandoulakis, Elias Theodoropoulos, Stamatia Kokkali, Chrysanthi Manolaki, Dimitra Iosifina Papageorgiou, Charis Birtsou, Aristofanis Paganas, Panagiotis Stachteas, Nikolaos Vlachopoulos, Ilias Pagkozidis, Akis Zeimbekis, Violeta Roka, Anastasios Giakoumis, Marina Kotsani, Ioanna Avakian, Efthymia Makridou, Magda Gavana, Anna-Bettina Haidich, Christina Avgerinou

**Affiliations:** 1 Laboratory of Primary Health Care, General Practice and Health Services Research, Aristotle University of Thessaloniki, Thessaloniki, Greece; 2 1st TOMY Serron, Prefecture of Serres, Greece; 3 Avdira Health Center, Prefecture of Xanthi, Greece; 4 Vari Health Center, Prefecture of Attica, Greece; 5 Pirgi Health Center, Prefecture of Chios, Greece; 6 Nea Kallikratia Health Center, Prefecture of Halkidiki, Greece; 7 Rodolivos Health Center, Prefecture of Serres, Greece; 8 Aristotle University of Thessaloniki Primary Health Care Research Network, Thessaloniki, Greece; 9 Regional Practice of Lagadikia, Zagliveri Health Center, Prefecture of Thessaloniki, Greece; 10 Litochoro Health Center, Prefecture of Pieria, Greece; 11 251 Hellenic Air Force General Hospital, Athens, Greece; 12 Kalloni Health Center, Prefecture of Lesvos, Greece; 13 Farkadona Health Center, Prefecture of Trikala, Greece; 14 Kato Achaea Health Center, Prefecture of Achaea, Greece; 15 Université de Lorraine, CHRU-Nancy, Pôle “Maladies du Vieillissement, Gérontologie et Soins Palliatifs”, F-54000 Nancy, France; 16 Regional Practice of Zappeio, Farsala Health Center, Prefecture of Larissa, Greece; 17 Laboratory of Hygiene, Social and Preventive Medicine, and Medical Statistics, Aristotle University of Thessaloniki, Thessaloniki, Greece; 18 Department of Primary Care and Population Health, University College London, Royal Free Campus, Rowland Hill Street, London, NW3 2PF UK

**Keywords:** Primary health care, Covid-19, Pandemic, Management, Greece

## Abstract

**Background:**

The coronavirus outbreak (COVID-19) tested health care systems worldwide. This qualitative study aimed to explore and understand the experiences, beliefs and concerns of Primary Care Professionals (PCPs) regarding the preparedness and response of primary care to the first wave of the pandemic in Greece, a country where a public structured primary care system has been developing.

**Methods:**

We conducted semi-structured telephone interviews with 33 PCPs (General Practitioners, community General Internal Medicine Specialists, community Paediatricians and nurses) recruited from all regions of Greece after the first wave of the pandemic (June 2020). Interviews were transcribed verbatim, data were anonymised and analysed. Thematic analysis was applied developing a conceptual framework.

**Results:**

Four main themes were identified: a) Primary care unit adaptation and issues faced during the pandemic; b) Management of suspected COVID-19 cases; c) Management of non-suspected cases; d) Consequences of the pandemic. In the first phase of the pandemic, remote management of suspected cases and their referral to the hospital were preferred as a result of a shortage of personal protective equipment and inaccessibility to coronavirus testing in primary care. Due to the discontinuation of regular medical services and the limited in-person contact between doctors and patients, chronic disease management and prevention programmes were left behind. Social and emotional consequences of the pandemic, such as workplace stigma, isolation and social seclusion, deriving from fear of viral transmission, as well as burnout symptoms and exhaustion were commonly experienced among PCPs. Positive consequences of the pandemic were considered to be the recognition of the importance of an empowered public healthcare system by citizens and the valuable insight, knowledge and experience professionals gained in times of crisis.

**Conclusions:**

Primary care has a key role to play during and after the pandemic by using its information infrastructure to identify at-risk groups, detect new cases of COVID-19, provide care according to needs, and carry out vaccination programmes. Central coordination and empowerment of primary care will increase its effectiveness, via public awareness, holistic patient management, and unburdening of hospitals.

## Background

Primary Care (PC) is the backbone of every health system. Experience from previous epidemics highlights the substantial role of PC and mandates the engagement of Primary Care Professionals (PCPs) in decision-making procedures [1, 2]. The continuation of PC services during the COVID-19 pandemic was deemed vital from the outset and countries were provided with guidance on PC services’ operation by the World Health Organisation (WHO) [3].

A strong PC system is the foundation of an efficient and effective national health service that eliminates inequalities in healthcare access [4]. In countries with limited access to PC, SARS-CoV-2 spread with greater speed and intensity [5]. On the other hand, countries with strong PC systems, such as Cuba and Australia, have succeeded in managing cases and relieving the burden of secondary and tertiary care [1, 6–8].

According to a monitoring instrument developed in the PHAMEU (Primary Health Care Activity Monitor for Europe project, Greece is among the countries with a relatively weak PC system [9]. The Greek National Health System (GNHS) is organised around Secondary Care (SC), with PC units remaining without systematic organisation and funding [10]. More specifically, public-funded PC in Greece consists of rural health centres and smaller rural regional practices which were founded in the 1980s, and more recently urban health centres and health units (called ‘TOMY’) which were founded or upgraded from pre-existing structures as part of a PC reform in 2018. Notably, the private PC health sector has traditionally been very popular in Greece, compared to other European countries with a longer history of established and organised public-funded PC. There is a large number of physicians running their own surgeries in the community, some of whom have affiliations with the social security system by which they are reimbursed for their services, although the majority of them work privately being paid directly by patients. These PC private surgeries are run by General Practitioners (GPs) and General Internal Medicine (GIM) specialists serving duties of a family physician, and Paediatricians looking after children.

Despite the organisational weaknesses of PC, Greece was considered a successful example in combating the first wave of the COVID-19 pandemic. The first patient signalled the beginning of the outbreak on 26.2.2020. On 23.3.2020, with a total of 624 confirmed cases and 15 deaths [11], a lockdown was announced [12], as available data at the time highlighted its effectiveness in reducing both viral transmission and mortality rates [13]. With the number of cases remaining in low levels, Greece partially lifted restrictions on 4.5.2020, and as of 9.6.2020 the WHO evaluated the country with Level 4 Status in COVID-19 Preparedness and Response [14].

In light of the SARS-CoV-2 pandemic, mapping out operations, challenges, limitations, dysfunctional aspects, and areas of improvement within a health system is of utmost importance. Such efforts to systematically break down, analyse, and address key issues and challenges are scarce in the literature. Hence, this study aimed to explore and understand the experiences, beliefs, and concerns of PCPs regarding the PC response to the pandemic in Greece during the first wave of COVID-19. The ultimate objective of the study was to draw useful conclusions on how to improve PC management and preparedness in the anticipated subsequent waves.

## Methods

### Design and setting

The study design was qualitative using semi-structured interviews with PCPs. The study population consisted of General Practitioners (GPs), community General Internal Medicine Specialists (GIMS), community Paediatricians and nurses, who were employees of public and private sector PC services in both rural and urban settings of the mainland and islands of Greece.

### Recruitment and sampling

Recruitment was facilitated by the Aristotle University of Thessaloniki Primary Health Care Research Network (AUTH.PHC.RN). Professionals were invited to take part in the study by email, purposively sampled according to the following selection criteria: a) GPs / GIMS / Paediatricians / nurses, working in PC; b) males / females; c) working in public or private sector; c) working experience more / less than 15 years; d) working in rural / urban areas; d) working in health centres / local health units / private practices; e) interested in taking part voluntarily (no remuneration provided); f) given written informed consent to participate. We concluded the interviews when data saturation was reached, i.e. no new themes emerged from the interviews.

### Data collection

A topic guide was developed prior to the study and reviewed by the research team. Main topics were: a) preparedness in the management of the pandemic [e.g. personal protective equipment (PPE), staff training]; b) staff shortages (pre-existing or during the pandemic); c) management of COVID-19 and non-COVID-19 emergencies and chronic diseases; and d) consequences of the pandemic.

Interviews were conducted remotely via telephone by PCPs/members of AUTH.PHC.RN, who were not acquainted to study participants, in June 2020 (the end of the delay phase of the first wave of the pandemic in Greece). All interviews were audio-recorded with consent, transcribed verbatim and identifiable data was anonymised.

### Data analysis

Transcripts were read by all members of the research team. Thematic analysis was used to identify key emergent themes and their meaning [15]. The analysis team identified a preliminary thematic framework. A coding framework was developed, agreed upon, and applied to all transcripts. The coding framework was applied to the transcripts and refined iteratively. Selected illustrative quotes are presented.

### Ethics approval and consent to participate

The study protocol received ethical approval by the Medical School Bioethics Committee of the Aristotle University of Thessaloniki (AUTH). All participants signed informed consent before inclusion in the study.

## Results

A total of 33 PCPs participated in the study, 24 doctors and 9 nurses, from 28 different health units (17 public and 11 private). A summary of participants’ characteristics is presented in Table 1.

**Table 1:** Participants’ characteristics

**Specialty**	N	Public n (%)	Private n (%)	Working Experience (< 15y) n (%)	Working Experience (15y or more) n (%)	Gender Male n (%)	Gender Female n (%)	Rural Area n (%)	Urban Area n (%)
PCPs (17 GPs, 1 GIMS)	18	10 (55.5)	8 (44.5)	8 (44.5)	10 (55.5)	9 (50.0)	9 (50.0)	9 (50.0)	9 (50.0)
Paediatricians	6	3 (50.0)	3 (50.0)	1 (16.7)	5 (83.3)	0 (0)	6 (100)	2 (33.3)	4 (66.7)
Nurses	9	9 (100)	0 (0)	3 (33.3)	6 (66.7)	0 (0)	9 (100)	3 (33.3)	6 (66.7)
**Total**	33	22 (66.7)	11 (33.3)	12 (36.4)	21 (63.6)	9 (27.3)	24 (72.7)	14 (42.4)	19 (57.6)

Four main themes emerged: a) Primary care unit adaptation and issues faced during the pandemic; b) Management of suspected COVID-19 cases; c) Management of non-suspected cases; d) Consequences of the pandemic.

### Primary care unit adaptation and issues faced during the pandemic

All PC units [except for Regional Practices (i.e. small GP practices serving rural areas) that ceased operations] followed a system of triage, distinguishing patients without suspected symptoms from those with COVID-19 symptoms.

*“When someone calls me and asks for an examination, I will ask him what this is about. If there is a fever or any small increase in temperature, I will make sure to examine him at the end of my shift.”* Participant 16, Private sector, General Practitioner

Units with multiple entrances used the main entrance for triage and a secondary entrance for emergencies. Triage was mainly performed by nursing staff according to guidelines and protocols of the National Public Health Organisation (NPHO/EODY), Regional Health Authorities (RHA/YPE), and the Ministry of Health.

*“Regarding the operations in our Health Centre, we have an entrance for the outpatient department, where citizens and personnel are checked and their temperature is taken. Another separate entrance for emergency cases and patients brought in via ambulance is also available. In the outpatient department entrance, health documents are collected, repeat prescription is performed and all documents are handed over to patients. Only if there is a need for examination, patients enter the Health Centre having an appointment, in order to avoid congestion.”* Participant 23, Public sector, Nurse

In several public units, a separate special examination and isolation area was set up either within or outside the Unit through the deployment of isolation booth structures. In the private sector, where separate examination rooms were not available, suspected COVID-19 cases were triaged by telephone. When these patients were examined face to face, there was a reasonable time difference from the rest of the appointments, taking place either at the beginning or at the end of working day, to prevent congestion in the waiting room and to allow time for disinfection and ventilation of the premises.

*“I think the biggest issue was how patients would visit us. Attendances, in the form of appointment, were taken care of with much greater accuracy. We could then avoid meetings within the practice, and there was time to decontaminate the room from one patient to the other.”* Participant 32, Private sector, Paediatrician

Health units faced detrimental shortages in PPE, such as surgical and high protection FFP2/FFP3 masks, gloves, face shields, bodysuits, and foot protection.

*“They were sending us little, very little equipment. We needed to record the slightest consumption of equipment, whether they were masks, antiseptics or other. We didn’t have uniforms,…, glasses. During the previous SARS [outbreak], a few years ago, they had sent us 4–5 uniforms and we have kept them. We had to manage with them for several months. We were afraid that, if anything came up, we only had this equipment to use.”* Participant 19, Public sector, Nurse

In the public sector, the provision of PPE from RHA/YPE was initially limited and then gradually increased in the majority of units. Many PCPs reported that they became equipped with masks and gloves at their own expenses, whilst important donations from public authorities, private companies, associations, and individuals were welcome.

*“The municipality gave us a container with two indoor spaces. We could examine patients and, of course, after each suspected case, the municipality was decontaminating the area.”* Participant 5, Public sector, General Practitioner

In the private sector, the acquisition of PPE was at the expense of PCPs, with Medical Associations across the country providing inadequate quantities, without aid by the government.

*“The truth is that we got charged a lot of money to provide ourselves with disinfectants [for our practices]; because the prices became too high, the products were too expensive, and as a result we paid too much for hygiene products”.* Participant 33, Private sector, Paediatrician

Moreover, the global shortage of PPE for citizens made PCPs’ efforts in securing adequate PPE supply a difficult task.

*“We had a big problem in finding face masks, gloves, light uniforms and waterproof uniforms, which are hard to find and of course extremely expensive”.* Participant 16, Private sector, General Practitioner

No major problems in personnel capacity were reported in the majority of public units, although in some cases PC staff were temporarily transferred to SC. In the private sector, on-site working hours were reduced. Yet, telephone consultations for patient information and support took over face-to-face patient contact.

*“We had mothers who had to stop working. However, we made ends meet. All of them got rotating special purpose leave. They understood that having continuous leave was not an option, one could not leave work for 2 or 3 months while the pandemic is looming. They were taking for example 6 days of special-purpose leave and 2 days of personal leave, which makes a total of 8 days within a month, that mothers took on rotation.”* Participant 5, Public sector, General Practitioner

Private doctors felt less supported in terms of education and information, and perceived guidelines as inundating, ever-changing, and confusing.

*“The anxiety of that first week, the stress about how I would get organised, which cases I would see was my main worry, that week was the first when we were reading different things from the paediatric association, different from the [regional] association, … different guidance from the government. That week there was a lot of confusion.”* Participant 31, Private sector, Paediatrician

On the contrary, the majority of employees in public units reported that they had received guidelines from RHA/YPE and took part in educational activities, such as the proper use of PPE.

*“We were informed by EODY [i.e. National Public Health Organisation] official website and from what was sent to us by Regional Health Authorities. Starting from there, we were browsing through websites, official sites, information sources from abroad searching for things to notice, such as atypical symptoms (dry mouth, diarrhoea).*” Participant 10, Public sector, General Practitioner

*“We had to learn how and if we had to use PPE. In this direction, hospitals helped a lot, by 1.5-h seminars for all healthcare workers to participate in. Then these participants were demonstrating [the use of PPE] to others in the health centre and we reached a very good point of understanding at this moment.”* Participant 5, Public sector, General Practitioner

### Management of suspected COVID-19 cases

In PC settings, fever, cough, and dyspnoea were the main symptoms that raised suspicion of COVID-19. Yet, other non-typical symptoms were reported, including hypothermia, loss of smell, gastrointestinal disturbances, delirium, and falls. In children, diarrhoea was considered a non-typical symptom.

*“In the beginning, we only had respiratory symptoms [that were considered suspicious]. Then they told us that in the elderly, delirium and gastroenteritis could also be present.”* Participant 6, Public sector, General Practitioner

Management of suspected cases was mainly performed via telephone, including history taking, confirmation of symptoms, and contact with NPHO/EODY for guidance and testing procedures.

*“The first thing we did was taking a good history. Then if it was a case of fever, we initiated the isolation process of the patient and his/her close contacts. We informed EODY and we were given guidelines for potential testing. Communication with patients was happening on a daily basis.”* Participant 4, Public sector, General Practitioner

In some units, a registry of telephone follow-ups for at-home patients was in place.

*“We kept a record sheet in which we reported symptomatology and we gave telephone instructions on what to do. We kept our patients at home and set up a review by phone the next day or the day after. Most of them were mild cases and ended up just with instructions over the phone.*” Participant 10, Public sector, General Practitioner”

In deterioration of symptoms or general health, patients were referred to SC for further assessment and potential admission.

*“I refer him to the hospital, while simultaneously calling EODY. I either send him by his own [transport] means, wearing a mask and after I have called the hospital to inform them that this patient will arrive by car in 20 min, so that they expect him. We either follow this procedure or we call an ambulance. We usually advise them to use their own transport, as I consider it a luxury for such a case to go by ambulance.”* Participant 3, Public sector, General Practitioner

However, it appears that PCPs used their own personal judgment in the management of suspected cases. Their decision-making regarding face-to-face assessments was affected by a balance between uncertainty over diagnosis and perceptions about risks, and it often was a stressful and difficult process, as illustrated by the quotes below:

*“I could say that, at least personally, I partially disregarded EODY’s guidelines. They were saying at the beginning that in case of fever they should stay at home. That is something I could not do, as sometimes there is not only COVID-related fever but also fever from other causes. We had to examine them. We examined patients and told them to stay at home. If their condition was deteriorating or the fever was too high, then we were re-examining them.”* Participant 5, Public sector, General Practitioner

*“My private practice was practically closed but guidance was given via telephone. I was afraid as I did not know whether I was giving the right advice and if the patient’s situation was deteriorating, I would have kept him in his house without examination, without auscultation.”* Participant 16, Private sector, General Practitioner

Another important determinant of management of suspected cases included PCPs’ individual knowledge and longitudinal relationship with a patient.

*“If that is a patient I routinely follow-up or if it is a first-time patient. In the second case, I would ask him/her to visit my private practice immediately, when calling for medical consultation. In the case of patients that I have been following-up for years, if I estimate that the symptoms are mild, very mild, I will not ask them to visit my practice. We will talk again on the phone and decide what to do depending on the progress.”* Participant 17, Private sector, General Practitioner

Telephone management was the case not only for patients with suspected symptoms but also for those with other symptoms not related to COVID-19. Video calls or other remote technologies were not widely endorsed in the public sector, despite professionals’ positive views, mainly due to shortages of equipment and lack of experience. On the contrary, these technologies were broadly used by PCPs working in the private sector, and mainly by Paediatricians, who reported similar use of such means before the pandemic.

*“You can estimate patients’ condition remotely. This could be better performed via video call instead of a simple telephone consultation. In no case does this replace immediate medical examination, but it can help in a lot of cases.”* Participant 9, Public sector, General Practitioner

At the end of the first wave of the pandemic, sufficient supplies of PPE, reinstatement of face-to-face physical examination, and increased familiarity with referral procedures and remote management of patients via telephone were the main changes observed.

### Management of non-suspected cases

During the first wave of the pandemic, Emergency Department attendance due to acute symptoms and conditions not related to COVID-19 was lower than expected, and delayed care-seeking was reported.

“*People with pain were coming, people with urological problems, who were saying ‘I am in pain like this for 10 days’ … or with an orthopaedic issue … many serious problems … and they were telling us that … ‘I was in pain for 10 days and I waited, but I cannot wait any longer’ … or heart problems … arrhythmia patients… with atrial fibrillation telling me ‘I was patient, I knew my pill was not covering me anymore, I was taking Rhythmonorm [propafenone] and I wasn’t feeling covered’; everyone did whatever they could to refrain from using the hospital or any health unit.”* Participant 25, Public sector, Nurse.

Poor attendance was also reported for those suffering with chronic illnesses, mainly due to lack of access to premises and fear of potential viral contamination, with potential detrimental effects on chronic disease management.

*“There was no follow-up, except when major issues arised. Patients were not visiting but were rather contacting us through the phone … some were visiting our practices just for repeat prescriptions … most of patients were contacting us through the phone for repeat prescriptions. Management of chronic illness was left seriously behind … there was no management at all.”* Participant 8, Public sector, General Practitioner

In many public PC units, patients were contacting doctors by telephone, whilst the use of other remote means (video calls, online platforms) was mainly reported by private practitioners. Private and public doctors were in agreement that while telephone management of chronic illnesses was helpful during lockdown, it should be used as a contingency plan and not as regular practice. In nearly all units, attendance in outpatient clinics bounced back in pre-pandemic numbers after the end of the lockdown restrictions.

The process for repeat prescriptions was not affected in either the public or private sector. The vast majority of prescription requests were actioned via telephone. Repeat prescriptions were sometimes facilitated by pharmacists, regional authorities, and municipality officers, such as those involved in the “Help at Home” program, providing basic home care support for older people. The newly introduced remote electronic prescription system was reportedly used only by a few public professionals, and was not widely used by private clinicians including Paediatricians. A common barrier was the lack of technology literacy among older patients.

*“Electronic prescription was performed through telephone in our Health Centre. Social security number was given over the phone, or pharmacists and relatives were coming, bringing prescriptions. Nobody was entering our Health Centre.*” Participant 6, Public sector, General Practitioner

In units performing home visits before the pandemic, fewer visits were reported during the first wave of COVID-19 and these were restricted to non-suspected cases, although the in-person assessment might in fact reveal signs of COVID-19 where it was not expected.

*“Before the pandemic I did home visits … now, I try to minimise them … as you never know what you may find in a home visit. In the beginning of the pandemic, I was contacted through the phone in the early morning and went on to visit an older lady I knew, who had dizziness and nothing more. She was in fact feverish, had an 80% oxygen saturation and after contacting EODY, she was transferred to the hospital by ambulance…”* Participant 13, Private sector, General Practitioner

Private GPs and GIMS avoided them, while some Paediatricians did home visits regularly, mainly for infant vaccination purposes.

*“Home visits increased…, mainly as parents did not want to visit the practice, fearing … even with appointments … that they might contract something more, they wanted to stay at home for most of the time and have the doctor visit them instead. For newborns, infants … I preferred to vaccinate in their homes so as not to expose them to the risks in private practice.”* Participant 33, Private sector, Paediatrician

### Consequences of the pandemic

The COVID-19 pandemic has had a tremendous impact on the GNHS, PCPs and citizens. Importantly, the need for an empowered public health system was acknowledged.

*“What we finally won, is a sense of worthiness in the eyes of the population, as in the last decades the Public Health System was depreciated. It was finally vindicated. Because, let’s face it, in difficult times, we all turn to it […the Greek National Health System]”.* Participant 12, Private sector, General Practitioner

More specifically, the need for an empowered PC system also emerged in view of gaps in care that became evident.

*“The need for general practitioners, family doctors, and their role was acknowledged and these doctors are the ones we did not have.”* Participant 17, Private sector, General Practitioner

Professionals gained valuable insight, knowledge, and experience in times of crisis.

*“I feel empowered, as I have the knowledge to manage cases and system’s operations in times of crisis. The Public Health System won knowledge in the management of cases and so did the Health Centre.”* Participant 28, Public sector, Paediatrician

Nevertheless, a cluster of participants in the study reported that the GNHS failed to be reinforced both in manpower and suitable equipment to support citizens’ needs in the occurrence of an anticipated second wave. Moreover, the pandemic signalled reduced activity or ceased operations in the country’s hospitals, skyrocketing the already long waiting list for routine appointments and procedures. Importantly, the GNHS reinforcement in the first wave was focussed on SC and the role of PC was not considered to be critical.

During this pandemic, PCPs gained clarity on infection prevention strategies, familiarised themselves with hygiene and safety protocols in the workplace, while showcased readiness and discipline in applying specific management guidelines. As a result, they gained invaluable experience in practising their profession under special circumstances, such as those of a pandemic.

On the other hand, loosening of strong patient-doctor relationships was reported, posing a threat to citizens’ participation in preventive medicine activities.

*“… I lost interpersonal contact with patients I did not know so well, I was trying to put everyone in order and make sure we are in an acceptable pre-symptomatic control state.”* Participant 4, Public sector, General Practitioner

Financial implications were reported mainly by professionals in the private sector, including loss of productive time due to delays of managing suspected cases in communication with NPHO/EODY and to longer appointment slots in order to avoid congestion and to secure airing and sanitising. Ultimately, the increased cost of securing PPE, the reduced income due to dropped attendance rates, as well as the inability for reimbursement in certain services, such as the newly introduced remote prescription system, posed an economic burden for private PCPs.

*“… Remote electronic prescription is definitely a practical measure for patients and for doctors when it comes to conserving paper. Our concerns are that there was never any plan for reimbursing private doctors for our services.”* Participant 27, Private sector, Internist

Social and emotional consequences of the pandemic were common among PCPs and citizens. A positive aspect was thought to be a sense of solidarity that was born through the crisis.

*“….They brought me things… masks, disinfectants and equipment, and that was very moving… ‘take care doctor’… I think that in such cases the world shows solidarity, especially to the person being in the frontline.”* Participant 14, Private sector, General Practitioner

Workplace stigma, isolation as well as social seclusion, deriving from fear of viral transmission, were mainly reported by nursing staff. Burnout symptoms as a result of stress at work and exhaustion were also reported.

*“I get tired of explaining all day to people that they cannot enter without wearing a mask. Some abide. Those who do not, are kind of pressuring me… I do not have the courage to fight with people and … sometimes I do not fight, but I feel like I am putting a burden, as if I have a problem and not others. It feels as if I have an issue in the end.”* Participant 18, Public sector, Nurse

A wide spectrum of mental health problems arising in the population amid the pandemic was reported by professionals, including withdrawal and lack of social interaction in children, and anxiety and depression, sometimes combined with suicidal ideation, in adults.

*“The economic aspect was also posing a detrimental role … the upside downs … a lot of people faced economic insecurities … and now lockdown, panic, personal responsibility, economic insecurity take over… everyone by himself… and some with minor [mental issues], that were not easily recognised … now these issues became evident… and such issues can not only be recognised, but they also have to be treated and heavily treated… with weekly visits to a Psychiatrist. The issue has become complex.”* Participant 12, Private sector, General Practitioner

## Discussion

The present qualitative study demonstrates the side-lining of PC in practice, as highlighted by the closure of Regional Practices as a result of shortages in personnel and isolated examination areas, the movement of PC medical and nursing staff towards SC reinforcement, the instructions of public health agencies calling for exclusive management of cases, as well as the availability of testing only in hospitals during the first wave.

Shortly after the start of the pandemic, the overwhelmed hospitals, the exponentially increased number of cases, and the experience gained from overseas management of the pandemic [1, 16–18] pushed the government to give PC an upgraded, more active role in managing suspected cases. Necessary PPE, shortages of which were globally observed mostly in PC settings [18–23], was secured and tests were given to health centres in affected areas, whilst PCPs adopted triage methods and remote management of mild cases.

In addition, several units used remote assessment and monitoring, as the literature demonstrates the contribution of telemedicine to the approach and management of both chronic and mild COVID-19 cases [24–26]. Awareness among PCPs in the use of telemedicine is necessary to ensure continued care and to maintain its quality [17, 23, 27–29]. However, professionals express reasonable concerns for the lack of physical contact and fear of the probability of missing important diagnoses, while patients sometimes find it difficult to adjust to a remote system of care and electronic means, so health inequalities may be aggravated [19, 20, 26]. Remote strategies also seem to augment examination time and increase professionals’ workload [18]. In total, telemedicine should be used for selected patients whose physical examination is not expected to alter their treatment plan, and as long as benefits outweigh the possible risks [30].

A large number of study participants reported concern over reduced prevention and management of chronic illness, due to exclusive engagement of the medical community with CΟVID-19 cases. Several studies confirm this phenomenon, even in countries with more robust PC, where the disruptions of prevention, treatment and rehabilitation of non-communicable diseases were described as an invisible epidemic [16, 21, 23, 29, 31, 32]. The period between consecutive waves of a pandemic is expected to secure the time needed to implement strategies targeted at prevention, triage for those in need, community education, and quality improvement of integrated health care services [22, 33, 34].

Ultimately, the psychological support of both PCPs and the general public is of utmost importance. The unprecedented changes to the commonly utilised patient management practices and to the working environments threaten their mental health and bring forward physical and mental exhaustion [35, 36]. Facilitating team meetings to achieve effective team functioning and enhanced performance under unprecedented and uncertain conditions, are described to be beneficial [18, 26]. As for individuals, the isolation and the rapid alterations in socioeconomic environment, due to the implementation of confinement measures, promote panic and fear phenomena, as well as the outburst of mental disorders [23, 30, 37–39]. The international scientific community emphasises the need for immediate detection and support for mental disorders, especially in vulnerable populations [8, 39, 40]. Primary care holds a crucial role in the identification, timely intervention and treatment of mental health conditions during and after COVID-19 [41].

Regarding the acclimatisation to the unprecedented conditions in the workplace, significant educational steps were taken after the first wave of the pandemic in Greece. Nevertheless, communication and information distribution between PCPs and national health organisations are often criticised in our interviews. The sense of lack of communication or miscommunication with public health agencies due to the constant modification to the provided guidelines is also reported in other studies [18, 26, 30]. Furthermore, prevention and health education should be similarly provided to the community in order to support the ongoing holistic management of the pandemic [23].

Globally, the prevalence of the COVID-19 pandemic requires coordinated efforts to achieve the goal of “flattening the curve”. Primary care practice is in this crisis very much community-oriented, contributing to limiting the spread of the infection [19, 33]. The contribution of PC in managing the pandemic through constant information and personalised care increases public awareness and compliance with isolation measures. Primary care has a key role to play during and after the pandemic by using its information infrastructure to identify at-risk groups, by providing comprehensive, continuous and person-centred care according to needs, by detecting new cases of COVID-19 and by tracing their close contacts, so as to reduce the risk of overwhelming hospital capacity [10, 23]. Central coordination and empowerment of primary care will increase its effectiveness, via public awareness, holistic patient management, and unburdening of hospitals.

### Strengths and limitations

The strength of this study is that it is, to our knowledge, among the first to explore the perspectives of frontline PCPs involved in the management of the pandemic. A qualitative approach including semi-structured interviews was used to achieve a level of understanding and interpretation of the associated operational conditions, dysfunctional aspects, and areas of improvement, which are rarely described in the currently available literature. Although we employed purposive sampling of participants, there is always a possibility some views and perspectives might not have been fully represented.

Another factor for consideration is that, since these interviews were conducted, the dynamic situation of the pandemic in Greece evolved, leading to a second wave, during which primary care was assigned a more central and proactive role in the handling of the pandemic. More specifically, PCPs were given direct access to COVID-19 testing for their patients, and most public PC units became vaccinating centres. Vaccinations against COVID-19 currently constitute a major part of PCPs’ workload. The PC workforce has been instrumental in delivering the vaccine at a mass scale and thus mitigating the pandemic. This transformation of PC during and after the second wave may have significant implications in terms of recognition of its importance in protecting health both at an individual and at a public health level.

## Conclusion

The COVID-19 pandemic redefined the role of PC, and PC units were quick to realise their potential. Important steps are yet to be taken for the management of the population’s health needs, with special focus on the systematic follow-up of COVID-19 patients at home, as well as the management of chronic conditions at times when social distancing is deemed necessary.

## Data Availability

The datasets generated during and/or analysed during the current study are not publicly available due to containing potentially identifiable information but may be available from the corresponding author on reasonable request, and subject to conforming to general data protection regulation.
